# Supplementary material for: The Crohn’s disease-related bacterial strain LF82 assembles biofilm-like communities to protect itself from phagolysosomal attack
Source: Commun Biol. 2021 May 25;4:627. doi: 10.1038/s42003-021-02161-7 (PMC8149705; doi:10.1038/s42003-021-02161-7)
Supplement: Supplementary file 2 — Supplementary Information [file 42003_2021_2161_MOESM2_ESM.pdf]

**The Crohn's disease-related AIEC strain LF82 assembles a biofilm-like matrix to protect intracellular microcolonies from phagolysosomal attack**

**Supplementary Information**

**Supplementary Figures**

**Supplementary Figure 1:** Analysis of the 500 LF82 genes presenting the most significantly changed mRNA fold at 1 h P.I. (**A)** and 6 h P.I. **(B)**. Gene annotations were manually curated to define 22 categories: Acid pH response, Biofilm matrix and regulation, Cell cycle, Central metabolism (carbohydrates, nucleotides, amino acids), Chemotaxis, Efflux pumps, Energy and respiratory metabolism, Import and export of nutrients, LF82 genes without homologue in the E. coli K12 genome, LF82 genes encoded by the mega plasmid pLF82, Membrane and envelope components, Metal homeostasis, oxidative stress, Phosphate homeostasis, SOS response, Diverse stress response (including cold shock proteins and phage shock proteins), Stringent response, Transcription factors, uncharacterized genes and putative virulence factors (presenting an homolog in the K12 genome). Numbers on the graph indicate the number of genes in the given category. **C)** Top panel, RNA-seq data from LF82 infecting THP1 macrophages for 6 h P.I. compared to liquid medium culture were analyzed according to regulon information. Regulon annotations were extracted from RegulonDB. For each transcription factor, the RNA-seq data from the regulated genes were collected, and only genes with a significant fold change (DEseq P-value $< 10^{-10}$) were considered. The box plot represents the median fold change of upregulated and downregulated genes from each regulon (bar), the distribution of 75% of the population (box) and outliers (cross). Bottom panel**,** RNA-seq data from LF82 infecting THP1 macrophages for 6 h P.I. compared to liquid medium culture were analyzed according to regulon information. The numbers of upregulated and downregulated genes from each regulon were plotted. **D)** Histogram showing the fold change in the expression of each LF82 gene belonging to the acidic pH response; biofilm and adhesins; flagella; glycolysis and ATP production annotation groups. For each gene, unfiltered RNA-seq data obtained at 1 h P.I. and 6 h P.I. were plotted.

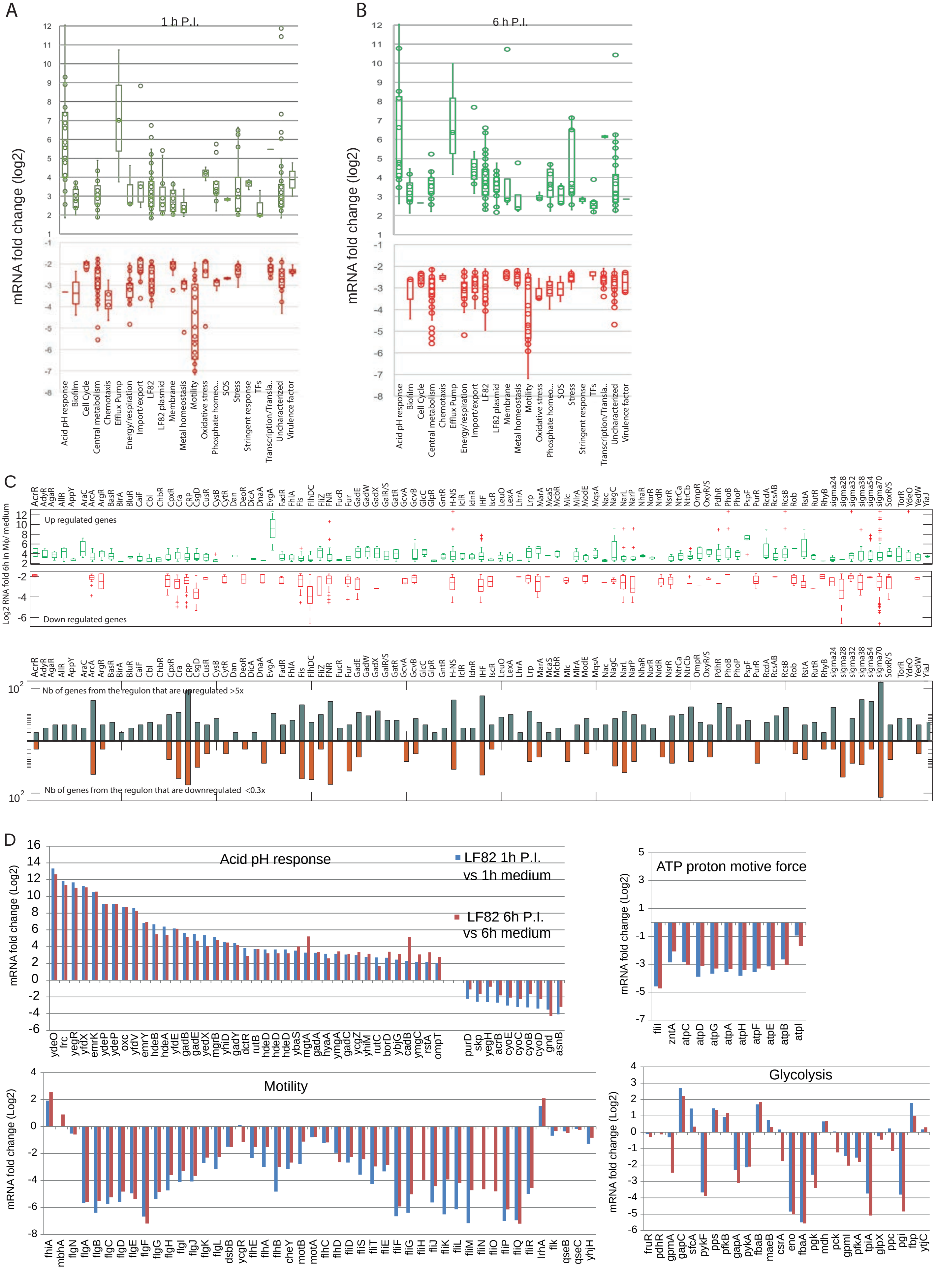

A
1 h P.I.
mRNA fold change (log2)
Acid pH response
Biofilm
Cell Cycle
Central metabolism
Chemotaxis
Efflux Pump
Energy/respiration
Import/export
LF82
LF82 plasmid
Membrane
Metal homeostasis
Motility
Oxidative stress
Phosphate homeo...
SOS
Stress
Stringent response
TFs
Transcription/Transla..
Uncharacterized
Virulence factor
B
6 h P.I.
mRNA fold change (log2)
C
Up regulated genes
Down regulated genes
Log2 RNA fold 6h in Mϕ/ medium
Nb of genes from the regulon that are upregulated >5x
Nb of genes from the regulon that are downregulated <0.3x
D
Acid pH response
LF82 1h P.I. vs 1h medium
LF82 6h P.I. vs 6h medium
mRNA fold change (Log2)
ATP proton motive force
Motility
Glycolysis

**Supplementary Figure 2: A)** RT-qPCR analysis of the induction of the biofilm pathway in Raw264.7 macrophages at 6, 18 and 24 h P.I.. **B)** Scatter plot of the LF82 mRNA fold change determined by RNA-seq at 6h P.I. in THP1 compared to 6h P.I. in Raw 264.7 macrophages. Only genes with a fold change above +1 and below -1 in Raw 264.7 macrophages are represented. **C)** Annotation of the biofilm formation pathways adapted from KEGG ko2026 according to RNA-seq data at 6h P.I. in Raw 264.7 macrophages vs planktonic exponential growth.

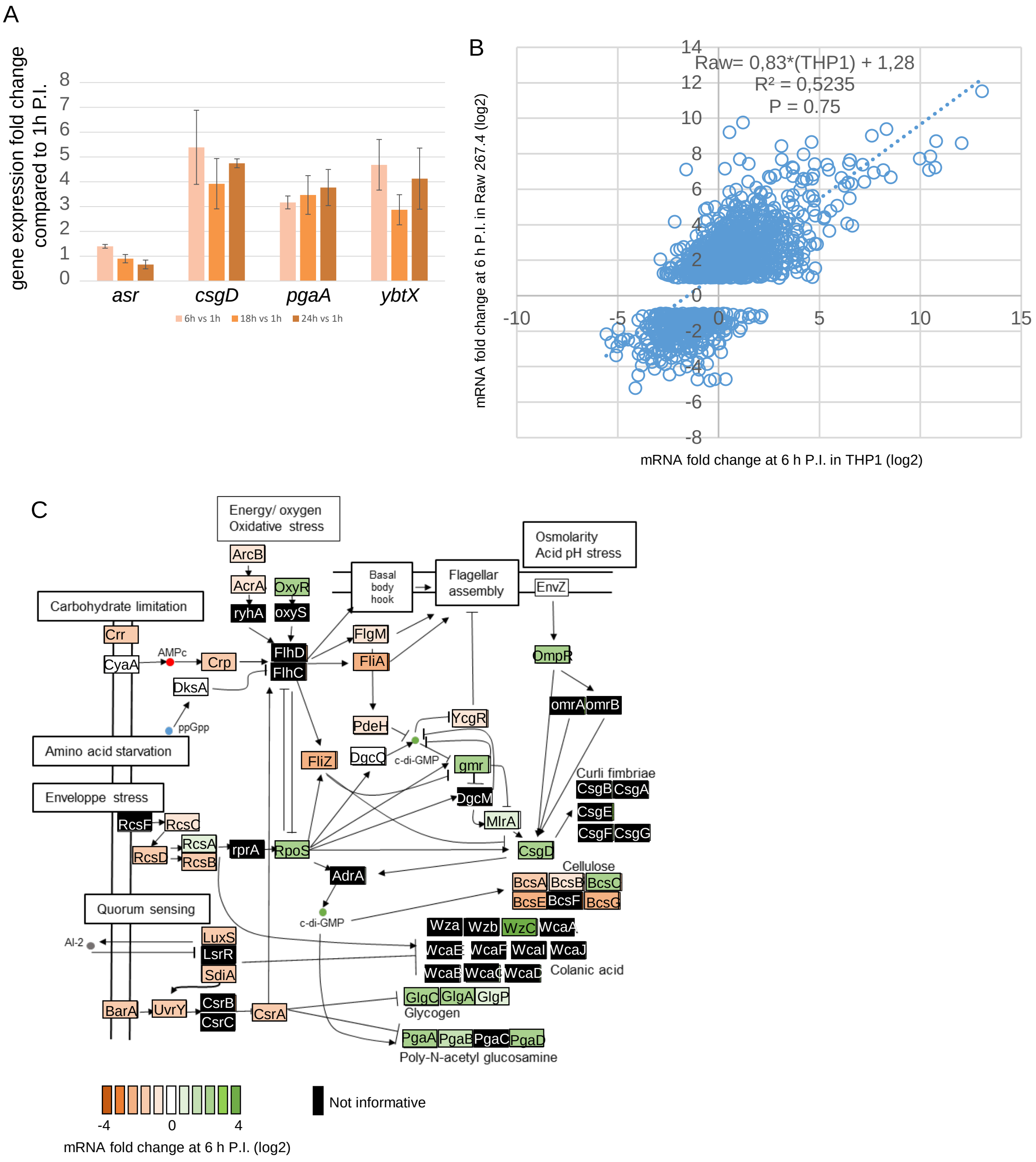


Supplementary Figure 2

**Supplementary Figure 3: Exopolysaccharide matrix is observed in three different types of macrophages** A) SBA labeling of the LF82 IBC at 1 h, 6 h and 24 h P.I. in Raw 264.7 macrophages. B) WGA labeling of the LF82 IBC at 1 h, 6 h and 24 h P.I. in HDMM macrophages. C) WGA labeling of the LF82 IBC in THP1 macrophages at 1 h and 24 h P.I. D) WGA labeling of the LF82 IBC in Raw 264.7 macrophages at 1 h and 24 h P.I. E) WGA labeling of the LF82 and LF82*csgD* IBC in Raw 264.7 macrophages at 72 h P.I.

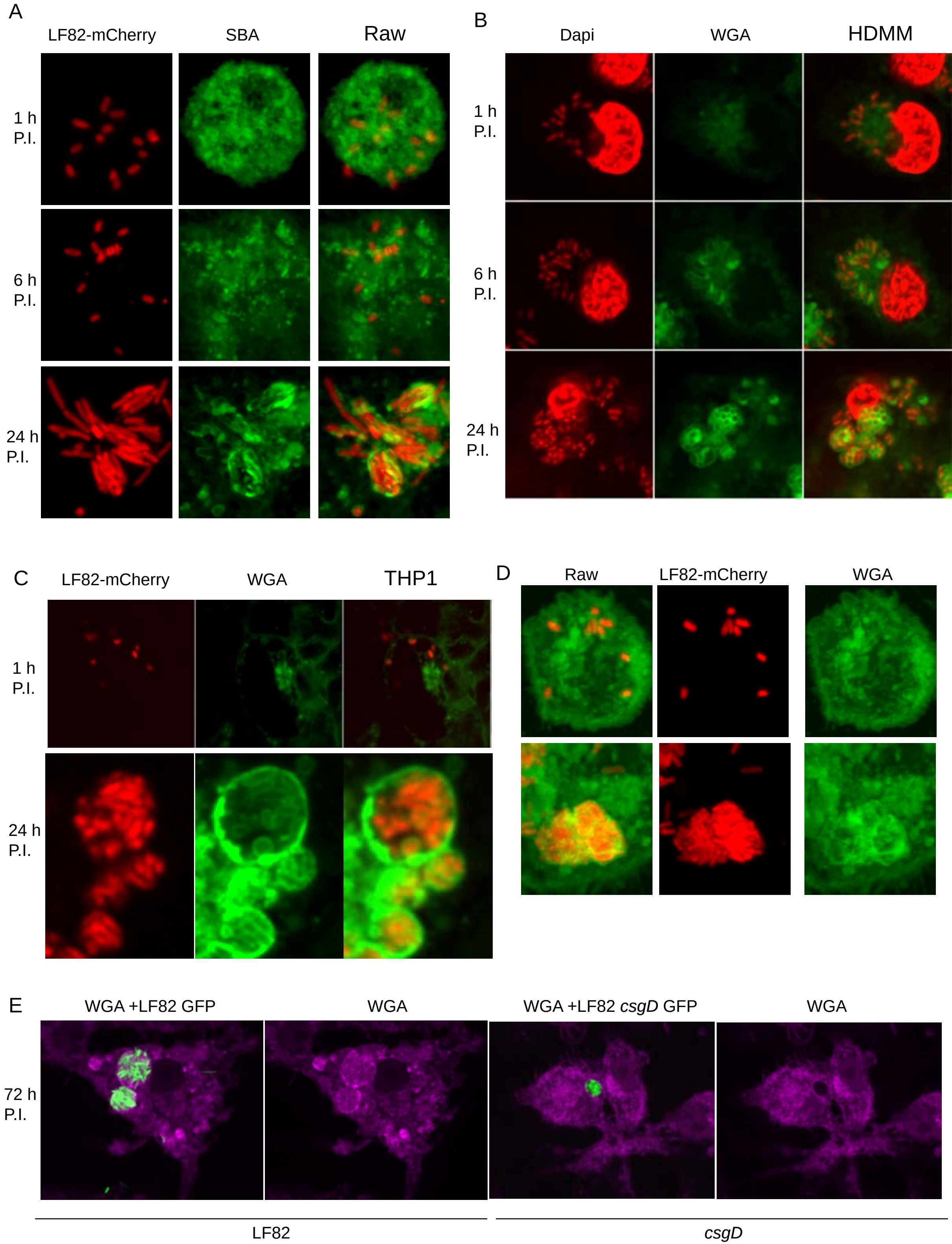
A
LF82-mCherry
SBA
Raw
1 h P.I.
6 h P.I.
24 h P.I.
B
Dapi
WGA
HDMM
1 h P.I.
6 h P.I.
24 h P.I.
C
LF82-mCherry
WGA
THP1
1 h P.I.
24 h P.I.
D
Raw
LF82-mCherry
WGA
E
WGA +LF82 GFP
WGA
WGA +LF82 *csgD* GFP
WGA
72 h P.I.
LF82
*csgD*

**Supplementary Figure 4: LF82 IBC did not present concanavalin A or Peanut Agglutinin (PNA) staining.** A) Concanavalin A labeling of Raw 264.7 macrophages infected or not by LF82. **B)** PNA labeling of Raw 264.7 macrophages infected by LF82.

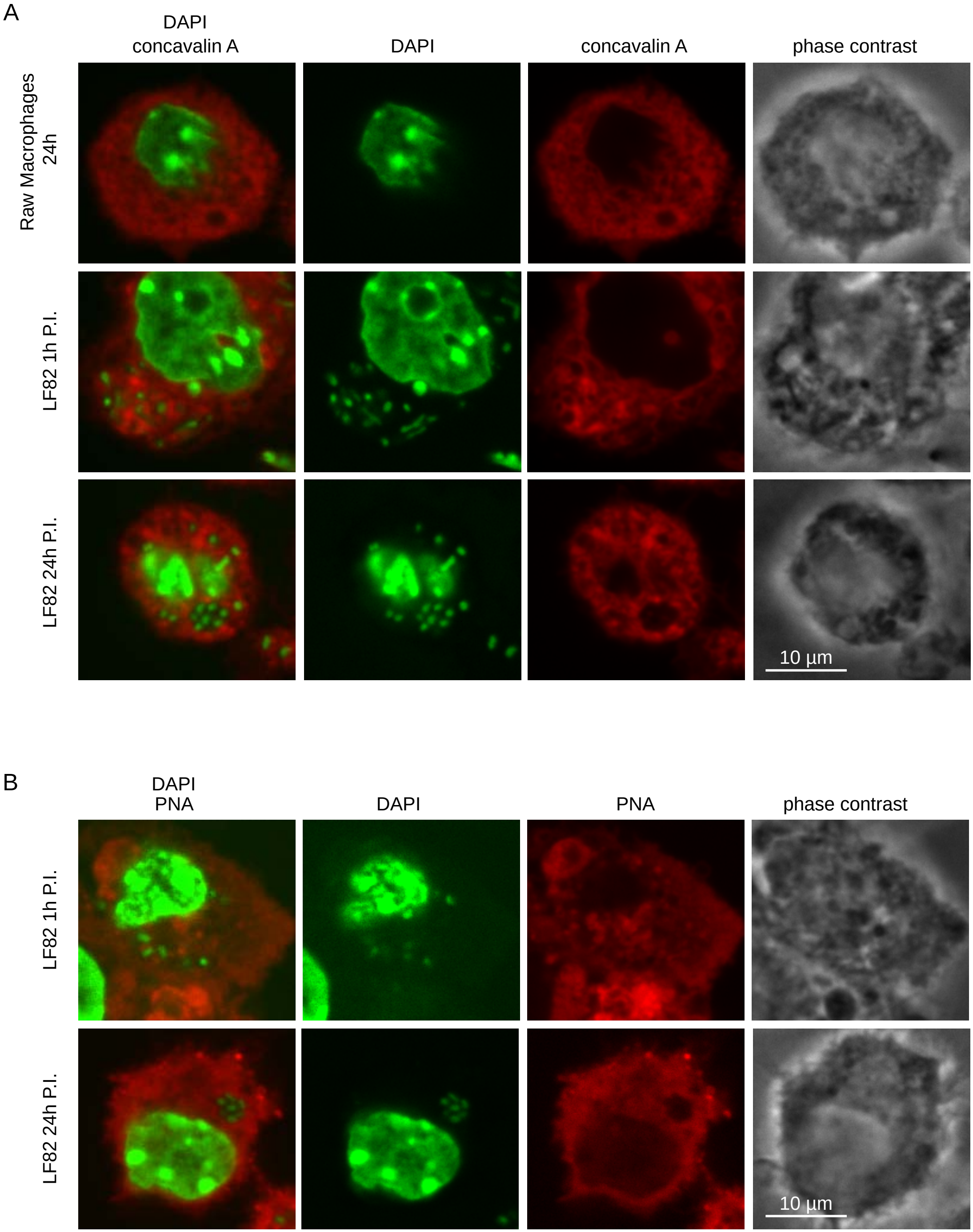
A
DAPI
concavalin A
DAPI
concavalin A
phase contrast
Raw Macrophages
24h
LF82 1h P.I.
LF82 24h P.I.
10 µm
B
DAPI
PNA
DAPI
PNA
phase contrast
LF82 1h P.I.
LF82 24h P.I.
10 µm

**Supplementary Figure 5: In vitro biofilm formation by LF82 and mutants on abiotic surfaces and Tn-seq calibration.** A) Measure of the Specific Biofilm Formation (*SBF*) indices by coloration of the wells of polystyrene microplate with crystal violet. LF82 WT and mutants were incubated for 24h at 37°C without agitation before washing and staining. B) Competition assays for LF82 mutants in co-infection with LF82 WT. The *recA, recO, umuC* and *rcsBD* mutants were co infected with LF82 at a MOI of 50 each. Raw 264.7 macrophages were lysed after 24h, live bacteria were counted by CFU and an aliquot was used for an overnight culture in LB to repeat infection the next day (2x24h). The same process was performed for a third round of infection (3x24h)

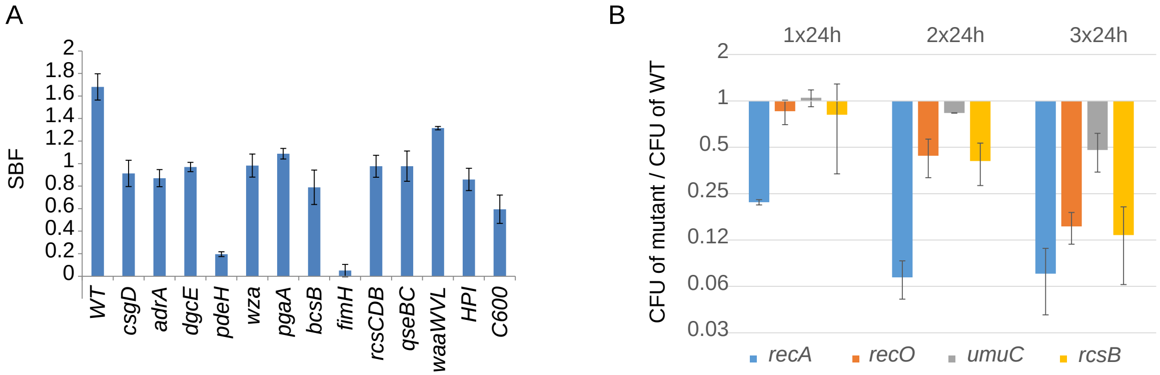

A
SBF
2
1.8
1.6
1.4
1.2
1
0.8
0.6
0.4
0.2
0
WT
csgD
adrA
dgcE
pdeH
wza
pgaA
bcsB
fimH
rcsCDB
qseBC
waaWVL
HPI
C600
B
1x24h
2x24h
3x24h
CFU of mutant / CFU of WT
2
1
0.5
0.25
0.12
0.06
0.03
recA
recO
umuC
rcsB


Figure S4

**Supplementary Figure 6: Tn-seq and RNA-seq results for different LF82 gene clusters.**

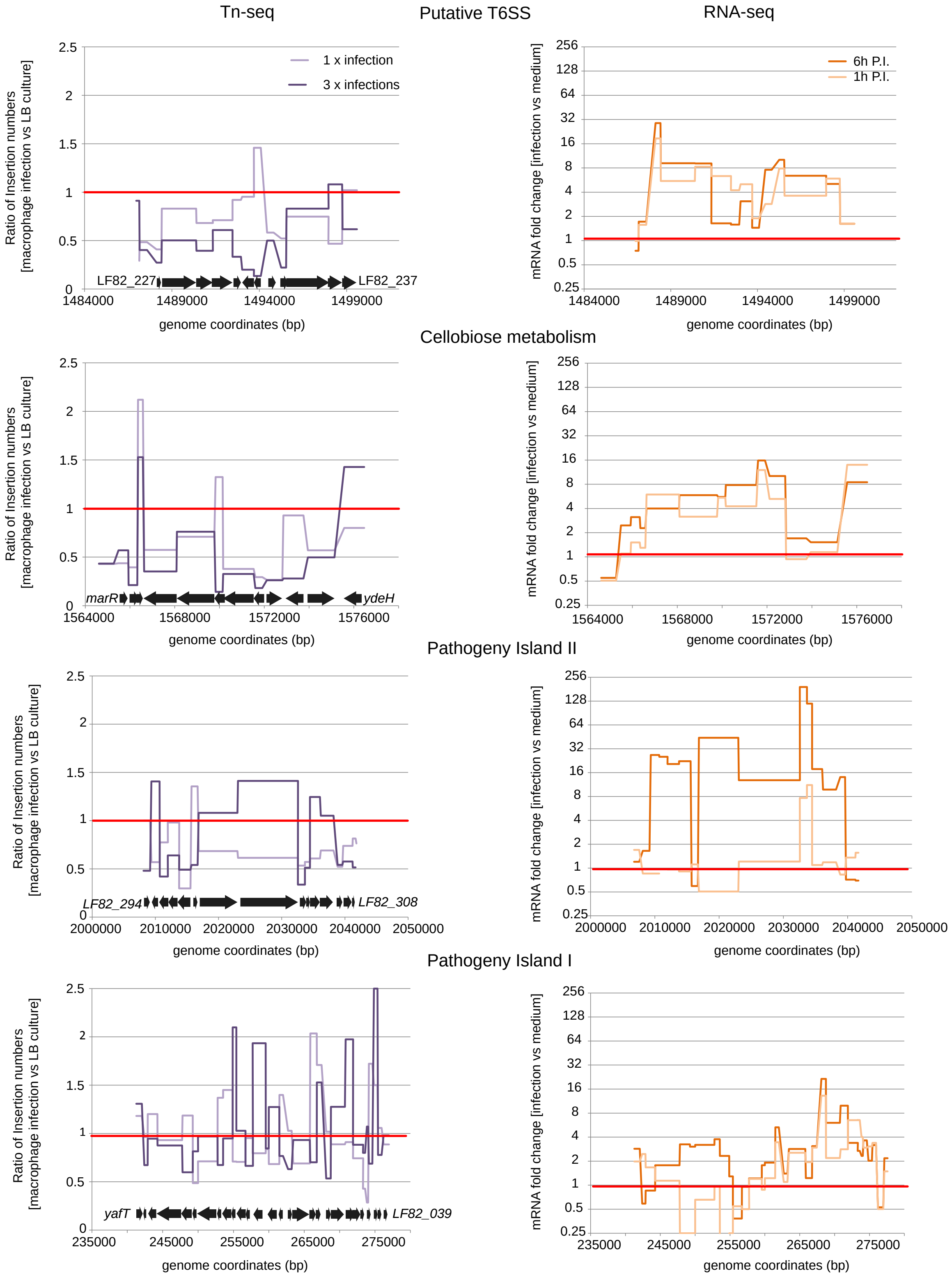

Tn-seq
Putative T6SS
RNA-seq
1 x infection
3 x infections
6h P.I.
1h P.I.
Ratio of Insertion numbers [macrophage infection vs LB culture]
mRNA fold change [infection vs medium]
genome coordinates (bp)
LF82_227
LF82_237
Cellobiose metabolism
marR
ydeH
Pathogeny Island II
LF82_294
LF82_308
Pathogeny Island I
yafT
LF82_039

**Supplementary Figure 7: Live imaging of LF82 mCherry P-HPI-GFP* expression during Raw 264.7 infection. A)** Snapshot of the field of view at 30 min and 1450 min P.I.**, B)** Montage on one selected Raw 264.7 macrophage presenting P-HPI-GFP* expression of a LF82's IBC. **C)** Montage on one selected Raw 264.7 macrophage that did not present P-HPI-GFP* expression of a LF82's bacteria. **D)** Quantification of P-HPI-GFP* expression over the infection kinetics. The green curve is the average of 10 IBC where visible P-HPI-GFP* expression was detected. The red-orange curves are examples of macrophage where no expression of P-HPI-GFP* was visible.

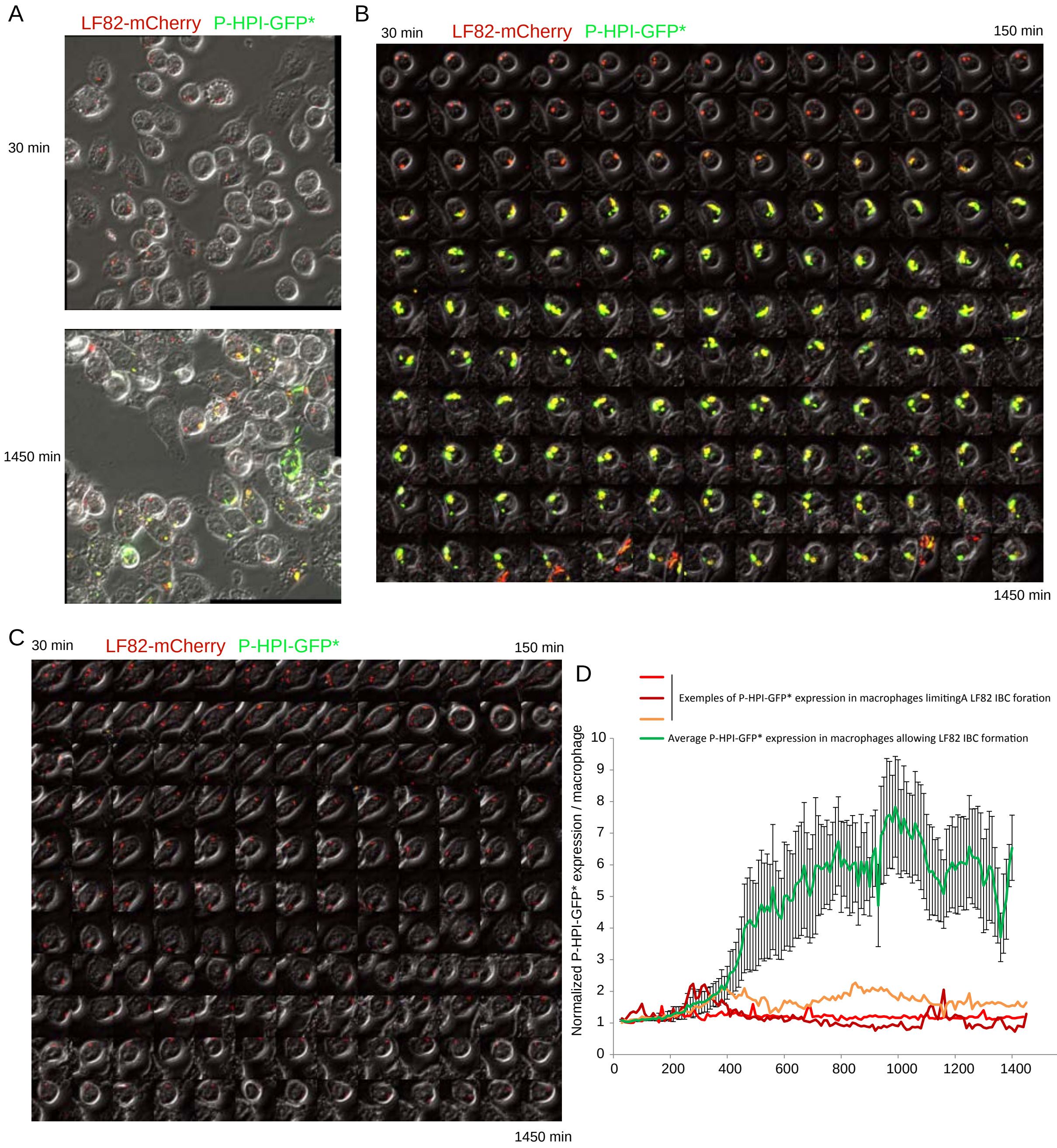
A
LF82-mCherry P-HPI-GFP*
30 min
1450 min
B
30 min
LF82-mCherry P-HPI-GFP*
150 min
1450 min
C
30 min
LF82-mCherry P-HPI-GFP*
150 min
1450 min
D
Exemples of P-HPI-GFP* expression in macrophages limitingA LF82 IBC foration
Average P-HPI-GFP* expression in macrophages allowing LF82 IBC formation
Normalized P-HPI-GFP* expression / macrophage
0
1
2
3
4
5
6
7
8
9
10
0
200
400
600
800
1000
1200
1400

**Supplementary Figure 8: HPI expression in Raw 264.7 and THP1 macrophages. A)** Comparison of the mRNA fold change of the HPI genes after infection of THP1 or Raw 264.7 macrophages compared to the expression in medium. Source data are presented on Supplementary Data 2 (windows THP1 vs Raw comparison). B) Montage on one selected THP1 macrophage presenting P-HPI-GFP* expression of a LF82's IBC.

A

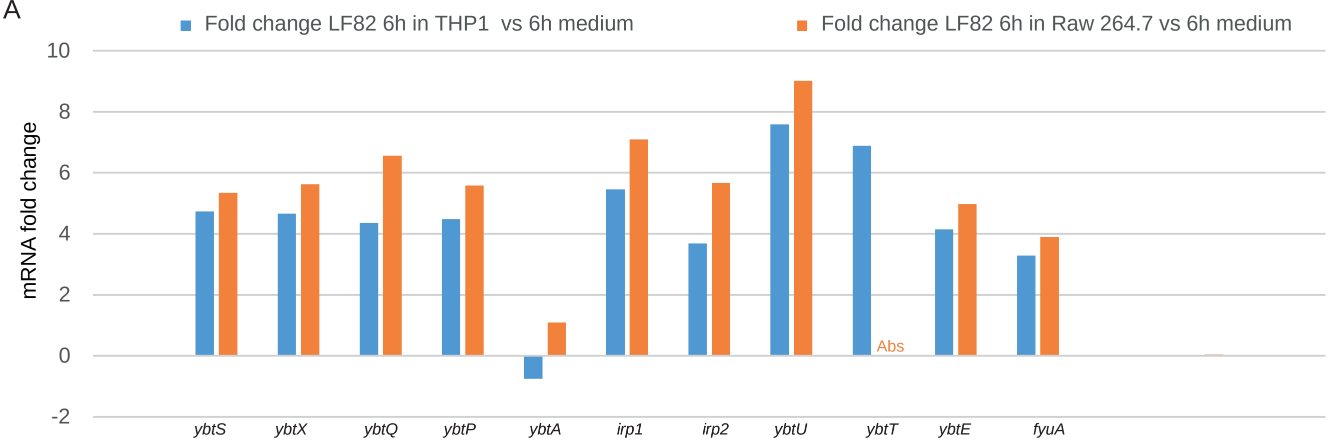

Fold change LF82 6h in THP1 vs 6h medium
Fold change LF82 6h in Raw 264.7 vs 6h medium
mRNA fold change
10
8
6
4
2
0
-2
Abs
ybtS
ybtX
ybtQ
ybtP
ybtA
irp1
irp2
ybtU
ybtT
ybtE
fyuA


B

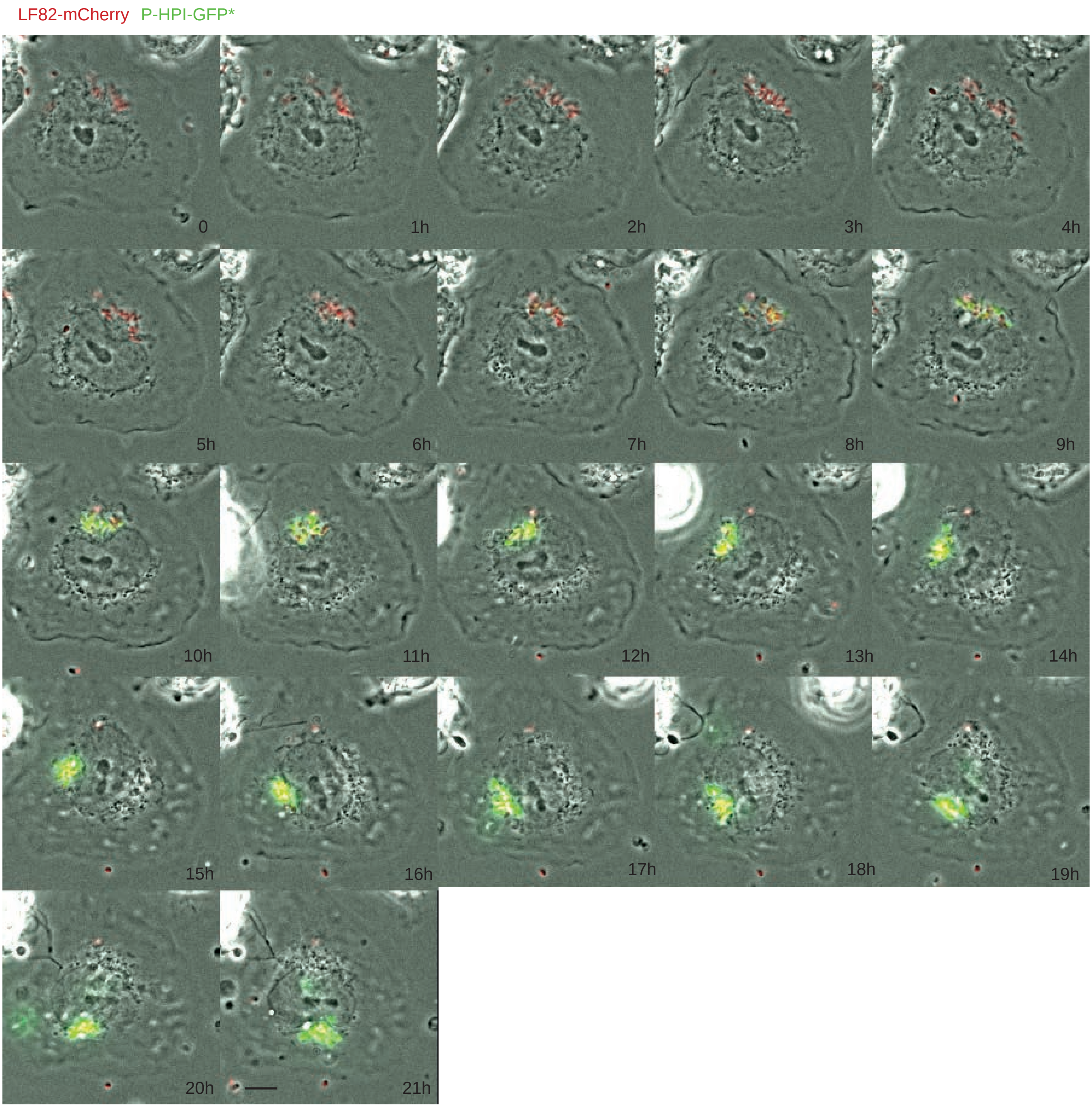

LF82-mCherry
P-HPI-GFP*
0
1h
2h
3h
4h
5h
6h
7h
8h
9h
10h
11h
12h
13h
14h
15h
16h
17h
18h
19h
20h
21h

**Supplementary Methods**

**Cell lines and Peripheral blood monocyte-derived macrophages**

THP1 (ATCC® TIB-202) monocytes (5x$10^5$ cells/ml) were differentiated into macrophages for 18 h in phorbol 12-myristate 13-acetate (PMA, 20 ng/ml) before infection. Peripheral blood monocyte-derived macrophages (MDM) were obtained from blood donors as previously described (Vazeille *et al,* 2015; Buisson *et al,* 2019).

**Live imaging**

Live imaging was performed on Raw 264.7 and THP1 macrophages infected with LF82 –mCherry pP-HPI-GFP*. Infection was performed in fluorodish (World Precision Instruments). Imaging was performed on an inverted Zeiss Axio Imager with a spinning disk CSU W1 (Yokogawa) equipped with an incubation chamber (Zeiss) and an Orca Flash camera.

**In vitro Biofilm assays**

Overnight cultures of LF82, LF82 mutants or K12 C600 E. coli were diluted 1/100 and 150µl were transferred to 96 wells polystyrene plates. Plates were incubated for 24h at 37°C without agitation. $OD_{600nm}$ of each well was recorded. Non adhesive bacteria were discarded. Biofilms were washed twice in 1x PBS and fixed with Bouin buffer (Sigma) for 1h at 60°C. Biofilms were washed twice in 1x PBS and stained with crystal violet for 20 min. Biofilms were washed twice in 1x PBS. Biofilm stained with crystal violet were eluted with 95% Ethanol for 30 min and the absorbance measured at $OD_{570nm}$ with a Tecan microplate reader. Biofilm formation is estimated by the SBF factor (Specific Biofilm Formation): SBF = ($OD_{570nm}$ sample - $OD_{570nm}$ control)/ $OD_{600nm}$. Data are average of three replicates +/- standard deviation.

**RNA-seq**

Macrophages were infected with exponentially growing LF82 at an MOI of 30. This MOI leads to a 90% infection rate scored at 1h P.I. by microscopy. Macrophages contains between 1 and 10 LF82, mean = 3. This procedure limits the proportion of bystanders macrophages in the population and presumably their contribution to the population transcriptome. Total RNA were extracted from THP1 macrophages infected by WT LF82 at 1 h and 6h P.I. Control experiments were performed with macrophages alone and bacteria alone cultivated for 1 h and 6 h in DMEM medium at 37°C and 5% $CO_2$ without agitation. RNA extraction was performed as described before (Demarre et al, 2019). Experiments were performed from biological duplicate. Human and bacterial rRNAs were depleted using the Ribo-Zero kits, then strand-specific libraries were constructed using the TruSeq Stranded Total RNA kit (Illumina). Paired-end sequencing (2 x 100 nt) was performed on a HiSeq 25000 sequencer. Reads alignment was performed using Bowtie 2 (Langmead & Salzberg, 2012) and significant fold changes accessed with the DE-seq pipeline (Anders & Huber, 2010). Fastq files were mapped with TopHat (v2.0.6), once on LF82 reference genome, with intron size limited to 5-20nt (options -i 5 & -I 20), and once on human reference genome hg19. Only uniquely mapping reads were kept (option -g 1). Count table were obtained with HTSeq. Alignment data are available on Table S1. RNA seq data for LF82 genome are available on Table S2 and on Table S6 for the Human genome.Total RNA were also extracted from Raw 264.7 macrophages infected by WT LF82 at 6h P.I. Experiments were performed from biological duplicate Samples were treated as described above. The small amount

of sequencing reads mapped to the genome of LF82 prevents DE-seq analysis, instead to collect insights for the most expressed genes we combined the two replicate and generated one mRNA fold change file available on Table S2. THP1 Data are available at GEO # GSE154648, Raw 264.7 were not of enough quality to be deposited to GEO, they are available upon request.

**Tn-seq**

Tn-seq was performed as described (Yamaichi & Dörr, 2017) with the exception that Mariner transposon's library was generated by electrotransformation of LF82 with the pTSC189Mariner vector instead of conjugation that is not efficient with LF82. About 2 million independent clones, corresponding to 20 electrotransformations, were collected. The library is diluted to an OD of 0.5 and 6 x 150 µl (≈ 4 x $10^7$ bacteria) were used to infect ≈ 6 x $10^6$ Raw 264.7 macrophages cultured in 6 x 2ml wells. After 24 h of infection macrophage were lysis buffer, bacteria were pelleted. Genomic DNA was immediately extracted from 2 wells and pulled together; this sample corresponds to the first round of selection (G1). Bacteria from the other wells were adjusted to OD = 0.01 in a 100 ml LB flask and grow overnight at 37°C. Bacterial cultures were diluted to an OD of 0.5 and immediately used to infect 4 wells of Raw 264.7 macrophages as previously. After 24 h of infection macrophage were lysis buffer, bacteria were pelleted. Genomic DNA was immediately extracted from 2 wells and pulled together; this sample corresponds to the second round of selection (G2). Bacteria from the last 2 wells were transferred to 100 ml LB flask and processed as described previously for the third round of selection (G3). In parallel the same library was cultured for 3 rounds in LB flask to access the selective pressure imposed by successive in vitro LB cultures. Genomic DNA was extracted at each step and processed for illumina sequencing as described in (Yamaichi & Dörr, 2017). Sequencing was performed at the Imagif sequencing facility on a miSeq Illumina sequencer. Sequencing data were aligned and analyzed with the Tn-seq explorer software (Solaimanpour *et al*, 2015). Tn-seq data are available on Table S4. The whole Tn-seq process, including the initial library preparation, was performed twice. We normalized the number of insertions sequenced for each experiment. At the genome level, we did not observe a statistically significant difference between replicates 1 and 2 (Pearson correlation = 0.79 - 0.88); therefore, we used the average of replicates 1 and 2 as indicative values.

**Antibodies**

| **Name** | **Source** | **Identifier** |
|---|---|---|
| Anti-LAMP1 antibody | abcam | AB25630 |
| Anti-LAMP1 antibody | abcam | AB25245 |
| CsgA | Gift from Matthew Chapman | (Zhou *et al*, 2013) |

**Bacterial Strains**

| **Name** | **Genotype** | **Reference** |
|---|---|---|
| AIEC LF82 | | (Glasser *et al.,* 2001) |
| AIEC LF82 bla | *ampC* | Gift from Nicolas Barnich |
| C600 | | Lab stock |
| AIEC LFVP74 | csgD::kan | This work |
| AIEC LFVP80 | glgC-A-P::kan | This work |
| AIEC LFVP81 | rcsC-B-D::kan | This work |
| AIEC LFVP86 | rseA-B::kan | This work |
| AIEC LFVP87 | waaW-V-L::kan | This work |
| AIEC LFVP88 | bcsB::kan | This work |
| AIEC LFVP89 | fimE::kan | This work |
| AIEC LFVP90 | adrA::kan | This work |
| AIEC LFVP91 | qseB-C::kan | This work |
| AIEC LFVP92 | pgaA::kan | This work |
| AIEC LFVP93 | gmr::kan | This work |
| AIEC LFVP94 | wza::kan | This work |
| AIEC LFVP95 | dgcE::kan | This work |
| AIEC LFAR01 | ybtS-X-Q-P-A-irp1::kan | This work |

**Plasmids**

| Name | Description | Antibiotic resistance | Reference |
|---|---|---|---|
| pKOBEGA | Recombineering vector | ampR specR | (Derbise *et al.,* 2003) |
| p*Prpsm-mcherry* | pGBM2-P*rpsM-mCherry* | specR | This work |
| pOM1-GFP | pGBM2-Pro3-GFP | specR | (Espéli *et al.,* 2001) |
| p1690-P-HPI-GFP* | ybtA and the promoter of irp1 colend SacI –XbaI in front of unstable GFP of the pSM1690 (Sternberg *et al*, 1999) | kanR | This work |

**Chemicals, Peptides, and Recombinant Proteins**

| Name | **Source** | **Identifier** |
|---|---|---|
| FluoProbes 647H - Phalloidin (653/675nm) | Interchim | FP-BZ9630 |
| FluoProbes 405 Phalloidin | Interchim | FP-1G6270 |
| WGA-alexafluor 647 conjuguate | Thermofisher | W32466 |
| WGA-alexafluor 555 conjugate | Invitrogen | W32464 |
| Concanavalin A, Alexa Fluor™ 647 Conjugate | Thermofisher | C21421 |
| Lectin PNA From Arachis hypogaea (peanut), Alexa Fluor™ 594 Conjugate | Thermofisher | L32459 |
| Lectin SBA From Glycine max (soybean), Alexa Fluor™ 647 Conjugate | Thermofisher | L32463 |
| Cis2 decenoic acid | Coger | CDX-D0249-M100 |
| gallium nitrate | SIGMA | 289892 |
| rh-M-CSF | Immunotools | 11343112 |

**Experimental Models: Cells and Cell lines**

| Name | **Source** | **Identifier** |
|---|---|---|
| Raw 264.7 | LGC | ATCC® TIB-71™ |
| THP1 | LGC | ATCC TIB-202 |
| Human Derived Macrophages from Monocytes (HDMM) | EFS Auvergne-Rhône-Alpes | Blood donors |

**Software**

| **Name** | **Source** | **Reference** |
|---|---|---|
| Matlab | https://fr.mathworks.com/ | |
| Tn-seq explorer | https://github.com/sina-cb/Tn-seqExplorer | (Solaimanpour *et al*, 2015) |

| | | |
|---|---|---|
| Fiji | https://imagej.net/Fiji | |
| Cytoscape | https://cytoscape.org/ | (Shannon *et al*, 2003) |
| Metamorph | https://fr.moleculardevices.com/ | |

**Supplementary References**


Anders S, Huber W (2010). "Differential expression analysis for sequence count data." Genome Biology, 11, R106.

Buisson A, Douadi C, Ouchchane L, Goutte M, Hugot JP, Dubois A, Minet-Quinard R, Bouvier D, Bommelaer G, Vazeille E, Barnich N (2019) Macrophages Inability to Mediate Adherent-Invasive E. coli Replication is Linked to Autophagy in Crohn's Disease Patients. Cells. 2019 Nov 5;8(11):1394

Derbise A, Lesic B, Dacheux D, Ghigo JM & Carniel E (2003) A rapid and simple method for inactivating chromosomal genes in Yersinia. *FEMS Immunol. Med. Microbiol.* **38:** 113–116

Espéli O, Moulin L & Boccard F (2001) Transcription attenuation associated with bacterial repetitive extragenic BIME elements. *J. Mol. Biol.* **314:** 375–386

Glasser AL, Boudeau J, Barnich N, Perruchot MH, Colombel JF & Darfeuille-Michaud A (2001) Adherent invasive Escherichia coli strains from patients with Crohn's disease survive and replicate within macrophages without inducing host cell death. *Infect. Immun.* **69:** 5529–5537

Langmead B, Salzberg S. (2012) Fast gapped-read alignment with Bowtie 2. Nature Methods., 9:357-359.

Shannon P, Markiel A, Ozier O, Baliga NS, Wang JT, Ramage D, Amin N, Schwikowski B & Ideker T (2003) Cytoscape: a software environment for integrated models of biomolecular interaction networks. *Genome Res.* **13:** 2498–2504

Solaimanpour S, Sarmiento F & Mrázek J (2015) Tn-Seq Explorer: A Tool for Analysis of High-Throughput Sequencing Data of Transposon Mutant Libraries. *PLOS ONE* **10:** e0126070

Vazeille E, Buisson A, Bringer MA, Goutte M, Ouchchane L, Hugot JP, de Vallée A, Barnich N, Bommelaer G, Darfeuille-Michaud A.Monocyte-derived macrophages from Crohn's disease patients are impaired in the ability to control intracellular adherent-invasive Escherichia coli and exhibit disordered cytokine secretion profile. J Crohns Colitis.9(5):410-20.

Yamaichi Y & Dörr T (2017) Transposon Insertion Site Sequencing for Synthetic Lethal Screening. *Methods Mol. Biol. Clifton NJ* **1624:** 39–49
