# Supplementary material for: The Crohn’s disease-related bacterial strain LF82 assembles biofilm-like communities to protect itself from phagolysosomal attack
Source: Commun Biol. 2021 May 25;4:627. doi: 10.1038/s42003-021-02161-7 (PMC8149705; doi:10.1038/s42003-021-02161-7)
Supplement: Supplementary file 3 — Description of Additional Supplementary Files [file 42003_2021_2161_MOESM3_ESM.pdf]

Description of Additional Supplementary Files

File name: Supplementary Data 1

Description: RNA-seq alignment data.

File name: Supplementary Data 2

Description: RNA-seq data for the LF82 genome.

File name: Supplementary Data 3

Description: GO analysis of LF82 RNA-seq data.

File name: Supplementary Data 4

Description: Tn-seq data for the LF82 genome.

File name: Supplementary Data 5

Description: Tn-seq data and GO analysis of the important and detrimental LF82 genes after 3 macrophage infections.

File name: Supplementary Data 6

Description: RNA-seq data for the human genome.

File name: Supplementary Data 7

Description: RNA-seq data for iron homeostasis genes in the human genome.

File name: Supplementary Data 8

Description: Source data underlying plots in main figures.
