# Supplementary material for: The Crohn’s disease-related bacterial strain LF82 assembles biofilm-like communities to protect itself from phagolysosomal attack
Source: Commun Biol. 2021 May 25;4:627. doi: 10.1038/s42003-021-02161-7 (PMC8149705; doi:10.1038/s42003-021-02161-7)
Supplement: Supplementary file 12 — Reporting Summary [file 42003_2021_2161_MOESM12_ESM.pdf]

nature research

Corresponding author(s): Olivier Espeli

Last updated by author(s): 2021/03/03

Reporting Summary

Nature Research wishes to improve the reproducibility of the work that we publish. This form provides structure for consistency and transparency in reporting. For further information on Nature Research policies, see our Editorial Policies and the Editorial Policy Checklist.

Statistics

For all statistical analyses, confirm that the following items are present in the figure legend, table legend, main text, or Methods section.

n/a	Confirmed	
☐	☒	The exact sample size (*n*) for each experimental group/condition, given as a discrete number and unit of measurement
☐	☒	A statement on whether measurements were taken from distinct samples or whether the same sample was measured repeatedly
☐	☒	The statistical test(s) used AND whether they are one- or two-sided<br>*Only common tests should be described solely by name; describe more complex techniques in the Methods section.*
☒	☐	A description of all covariates tested
☒	☐	A description of any assumptions or corrections, such as tests of normality and adjustment for multiple comparisons
☒	☐	A full description of the statistical parameters including central tendency (e.g. means) or other basic estimates (e.g. regression coefficient) AND variation (e.g. standard deviation) or associated estimates of uncertainty (e.g. confidence intervals)
☒	☐	For null hypothesis testing, the test statistic (e.g. *F*, *t*, *r*) with confidence intervals, effect sizes, degrees of freedom and *P* value noted<br>*Give P values as exact values whenever suitable.*
☒	☐	For Bayesian analysis, information on the choice of priors and Markov chain Monte Carlo settings
☒	☐	For hierarchical and complex designs, identification of the appropriate level for tests and full reporting of outcomes
☒	☐	Estimates of effect sizes (e.g. Cohen's *d*, Pearson's *r*), indicating how they were calculated

Our web collection on statistics for biologists contains articles on many of the points above.

Software and code

Policy information about availability of computer code

Data collection	Microscopy data were collected with Metaporph.
Data analysis	For this analysis we used<br>Matlab https://fr.mathworks.com/<br>Tn-seq explorer https://github.com/sina-cb/Tn-seqExplorer<br><br>Tn-seq explorer https://github.com/sina-cb/Tn-seqExplorer<br>Fiji https://imagej.net/Fiji<br>Cytoscape https://cytoscape.org/<br><br>for RNAseq<br>Reads alignment was performed using Bowtie 2 (Langmead & Salzberg, 2012) and significant fold changes accessed with the DE-seq pipeline (Anders & Huber, 2010). Fastq files were mapped with TopHat (v2.0.6), once on LF82 reference genome, with intron size limited to 5-20nt (options -i 5 & -I 20), and once on human reference genome hg19. Only uniquely mapping reads were kept (option -g 1). Count table for THP1 data were obtained with HTSeq, and count table for LF82 data were obtained using a custom R script, were each gene count is incremented when overlapped by a fragment.<br>Fiji https://imagej.net/Fiji<br>Cytoscape https://cytoscape.org/

For manuscripts utilizing custom algorithms or software that are central to the research but not yet described in published literature, software must be made available to editors and reviewers. We strongly encourage code deposition in a community repository (e.g. GitHub). See the Nature Research guidelines for submitting code & software for further information.

Data

Policy information about availability of data

All manuscripts must include a data availability statement. This statement should provide the following information, where applicable:

- Accession codes, unique identifiers, or web links for publicly available datasets
- A list of figures that have associated raw data
- A description of any restrictions on data availability

The datasets generated during and/or analysed during the current study are available from the corresponding author on reasonable request. RNA-seq datasets generated during and/or analysed during the current study are available in the GSE154648 repository GEO

Field-specific reporting

Please select the one below that is the best fit for your research. If you are not sure, read the appropriate sections before making your selection.

- [x] Life sciences
- [ ] Behavioural & social sciences
- [ ] Ecological, evolutionary & environmental sciences

For a reference copy of the document with all sections, see nature.com/documents/nr-reporting-summary-flat.pdf

Life sciences study design

All studies must disclose on these points even when the disclosure is negative.

Sample size	For imaging analysis between 100 and 2000 fluorescent objects were analyzed for each replicate. Between 3 and 7 independant experiments were performed.
Data exclusions	We did not exclude any data
Replication	RNA-seq vas performed in duplicate with independent bacteria and macrophage cultures. For imaging and CFU etween 3 and 7 independant experiments were performed.
Randomization	n/a
Blinding	n/a

Reporting for specific materials, systems and methods

We require information from authors about some types of materials, experimental systems and methods used in many studies. Here, indicate whether each material, system or method listed is relevant to your study. If you are not sure if a list item applies to your research, read the appropriate section before selecting a response.

Materials & experimental systems

n/a	Involved in the study	
[ ]	[x]	Antibodies
[ ]	[x]	Eukaryotic cell lines
[x]	[ ]	Palaeontology and archaeology
[x]	[ ]	Animals and other organisms
[x]	[ ]	Human research participants
[x]	[ ]	Clinical data
[x]	[ ]	Dual use research of concern

Methods

n/a	Involved in the study	
[x]	[ ]	ChIP-seq
[x]	[ ]	Flow cytometry
[x]	[ ]	MRI-based neuroimaging

Antibodies

Antibodies used	Anti-LAMP1 antibody ,abcam,A B25630. Anti-LAMP1 antibody, abcam, AB25245. CsgA Gift from Matthew Chapman (Zhou et al, 2013)
Validation	CsgA Gift from Matthew Chapman was validated by the Chapman group (Zhou et al, 2013). Anti-Lamp1 antibodies were validated by Abcam: https://www.abcam.com/lamp1-antibody-h4a3-ab25630.html

Eukaryotic cell lines

Policy information about cell lines

Cell line source(s)	Raw 264.7 LGC ATCC® TIB-71™, THP1 LGC ATCC TIB-202,
Authentication	none of the cell line were athentified
Mycoplasma contamination	cell lines were not tested for mycoplasma contamination
Commonly misidentified lines (See ICLAC register)	N/A
